# Reduced Myelin Signal in Normal-appearing White Matter in Neuromyelitis Optica Measured by 7T Magnetic Resonance Imaging

**DOI:** 10.1038/s41598-019-50928-0

**Published:** 2019-10-07

**Authors:** I-Jun Chou, Radu Tanasescu, Olivier E. Mougin, Penny A. Gowland, Christopher R. Tench, William P. Whitehouse, Bruno Gran, Esmaeil Nikfekr, Basil Sharrack, Gordon Mazibrada, Cris S. Constantinescu

**Affiliations:** 1 Division of Clinical Neuroscience, School of Medicine, University of Nottingham, Nottingham, UK; 2 Division of Academic Child Health, School of Medicine, University of Nottingham, Nottingham, UK; 3 Division of Paediatric Neurology; Chang Gung Children’s Hospital at Linkou, Chang Gung University College of Medicine, Taoyuan, Taiwan; 4 Division of Neurosciences, University of Medicine and Pharmacy Carol Davila, Department of Neurology, Colentina Hospital, Bucharest, Romania; 5 Sir Peter Mansfield Imaging Centre, School of Physics and Astronomy, University of Nottingham, Nottingham, England UK; 6 Department of Neurology, University of Sheffield, Sheffield, UK; 7 Department of Neurology, Queen Elizabeth Hospital Birmingham, Birmingham, UK

**Keywords:** Neuroscience, White matter disease

## Abstract

Whether the integrity of normal-appearing white matter (NAWM) is preserved in neuromyelitis optica spectrum disorders (NMOSD) is open to debate. To examine whether the tissue integrity of NAWM in NMOSD is compromised compared to that in healthy controls and patients with multiple sclerosis (MS), we prospectively enrolled 14 patients with NMOSD, 12 patients with MS, and 10 controls for clinical functional assessments and quantitative imaging, including T1 relaxation time (T1) and magnetization transfer ratio (MTR) at 7 Tesla. Cognitive performance on the Paced Auditory Serial Addition Test with a 3-second interstimulus interval (PASAT-3) was significantly lower in the NMOSD compared to the MS group (mean number of correct answers, 34.1 vs. 47.6; *p* = 0.006), but there were no differences in disease duration or disability. Histograms of T1 and MTR maps of NAWM demonstrated a decreased peak height in patients with NMOSD compared to the healthy controls, but not compared to patients with MS. Using 7T quantitative magnetic resonance imaging (MRI), this study showed that the NAWM in patients with NMOSD is abnormal, with reduced myelin signal; this was not previously observed using MRI at a lower field strength.

## Introduction

Neuromyelitis optica spectrum disorders (NMOSD) are rare but debilitating conditions with an estimated prevalence of 0.52 to 4.4 per 100,000 population^1^. Classic NMO is characterized by optic neuritis and longitudinally extensive transverse myelitis (LETM)^2^. NMOSD initially presents as a limited form of optic neuritis or LETM, whose diagnosis can be supported by a positive serological test of autoantibodies against aquaporin-4 (AQP4)^3^. The term NMOSD encompasses classic NMO and cases with or without autoantibodies against AQP4^4^. Approximately a quarter of AQP4 seronegative NMOSD patients have antibodies against myelin oligodendrocyte glycoprotein (MOG)^5^. NMOSD are associated with reduced quality of life and increased mortality.

Many patients with NMOSD exhibit normal brain magnetic resonance imaging (MRI) findings at diagnosis, despite the fact that cavitation, suggesting a necrotizing process, is frequently found histologically in lesions in the medulla oblongata, spinal cord, and optic nerves^6,7^. Whether normal-appearing white matter (NAWM) on imaging studies for NMOSD is indeed normal is still open to debate. The magnetization transfer ratio (MTR) of NAWM at 1.5 Tesla (T) did not differ between NMOSD and healthy controls^8,9^. The diffusion tensor imaging (DTI) at 3T revealed abnormalities confined to the optic and corticospinal tracts^10,11^. In contrast, patients with multiple sclerosis (MS) have multiple brain white matter (WM) lesions, where demyelination and remyelination is the characteristic histological finding^12^. Both lesions and NAWM in MS patients have been quantified using quantitative T1 mapping and MTR, with demyelination, inflammation, oedema and tissue disorganisation being assessed histopathologically^12–15^. In addition, at 7T, both quantitative T1 mapping and the MTR can detect changes within NAWM in clinically isolated syndromes and relapsing-remitting MS (RRMS) relative to healthy controls^16^ via imaging with submillimetre resolution. Quantitative imaging at 7T is capable of revealing the damaged substrate within WM lesions and NWAM in NMOSD.

Based on aforementioned MRI and histological findings, we hypothesized that (1) the extent of structural change of WM lesions in NMOSD is more severe than in MS, but that (2) the integrity of NAWM in NMOSD is less compromised than in MS. Therefore, we prospectively enrolled patients with NMOSD, patients with MS, and healthy participants and acquired brain MRI images at 7T, for comparison of the measured T1 relaxation time and MTR maps among the groups.

## Results

### Patient characteristics

We prospectively investigated 36 participants: 14 patients with NMOSD, 12 patients with MS, and 10 healthy participants. Ten (71%) of the patients with NMOSD had classic NMO (Supplementary Table 1). Eight (67%) of the patients with MS had RRMS, two (17%) had secondary progressive MS (SPMS), and two (17%) had primary progressive MS (PPMS).

The date of the MRI scan was separated from a clinical attack by at least 30 days. One of the patients with NMOSD was treated with corticosteroids alone, and the others received long-term immunosuppressants: five had azathioprine, five had rituximab, and three had mycophenolate mofetil; of these patients, three combined their treatment with low-dose corticosteroids. Four patients with MS received long-term immunomodulatory agents: one used interferon beta-1a, one glatiramer acetate, one natalizumab, and one dimethyl fumarate.

### Clinical assessments

Clinical characteristics and functional assessment results are summarized in Tables 1 and 2, respectively. There were no significant differences in disease duration, Expanded Disability Status Scale (EDSS) score, self-administered Fatigue Severity Scale (FSS) score, quantitative functional test results of leg function/ambulation (Timed 25-Foot Walk), or arm function (9-Hole Peg Test) between the patients with MS and NMOSD. However, cognitive function (Paced Auditory Serial Addition Test with a 3-second interstimulus interval, PASAT-3) was significantly lower in the patients with NMOSD than in MS patients (mean number of correct responses, 34.1 vs. 47.6, respectively; *p* = 0.006). The Multiple Sclerosis Functional Composite (MSFC) scores derived by summing the three aforementioned components were similar between the NMOSD and MS patients. The scores were significantly worse in NMOSD and MS patients than in controls except for cognitive function (PASAT-3), which was similar between the patients with MS and controls. In healthy controls, age was only negatively correlated with hand function score (rho = −0.665, *p* = 0.036).

**Table 1:** Comparisons of demographic and clinical characteristics between the patients with NMOSD, patients with MS and healthy controls.

	NMOSD (n = 14)	MS^a^ (n = 12)	Controls (n = 10)	P
Median age, years (IQR)	54.0 (37.0–63.0)	55.5 (40.5–63.5)	44.5 (24–57)	0.416
Female, n (%)	10 (71)	7 (58)	7 (70)	0.769
Presence of serum Ab, n (%)	NMO-IgG/AQP4: 8 (57) MOG: 1 (7)	n/a	n/a	
Median disease duration, years (IQR)	8 (3.0–11.0)	13 (7.5–20.5)	n/a	
Receiving immunosuppressants or immunomodulatory agents, n (%)	14 (100)^b^	5 (42)^c^	n/a	.

Abbreviations: Ab, antibody; AQP4, aquaporin 4; IgG: immunoglobulin G; IQR, interquartile range; MOG, myelin oligodendrocyte glycoprotein; MS, multiple sclerosis; NMO, neuromyelitis optica; NMOSD, neuromyelitis optica spectrum disorders; n/a, not applicable or not available; SD, standard deviation.

^a^Eight (67%) MS patients had relapsing remitting MS, two (17%) had secondary progressive MS, and two (17%) had primary progressive MS.

**Table 2:** Comparisons of EDSS, FSS, and MSFC between the 14 patients with NMOSD, 12 patients with MS, and 10 healthy controls.

	NMOSD (n = 14)	MS (n = 12)	Controls (n = 10)	P
**Median EDSS and FSS (IQR)**				
EDSS	5.25 (4.0–6.0)	6.0 (4.0–6.5)	n/a	
	n = 12	n = 12	n = 10	
FSS	5.6 (5.3–6.3)^A^	6.0 (4.9–6.4)^A^	2.1 (1.2–2.6)^B^	<0.001*
**Mean MSFC raw score and z-score** ^**a**^ **(±SD)**				
T25-FW^b^	n = 11	n = 12	n = 10	
Raw score, s	8.56 ± 2.46^A^	9.98 ± 4.14^A^	4.59 ± 0.30^B^	<0.001*
Z-score	−13.40 ± 8.31^A^	−17.69 ± 14.56^A^	0 ± 1^B^	<0.001*
9HPT	n = 14	n = 12	n = 10	
Raw score dominant hand, s	34.83 ± 24.79^A^	27.83 ± 4.80^A,B^	17.60 ± 1.93^B^	0.044*
Raw score nondominant hand^c^, s	86.48 ± 199.24	31.85 ± 12.10	19.95 ± 2.75	0.375
Z-score of both hands	−3.89 ± 2.00^A^	−3.42 ± 1.20^A^	0 ± 1^B^	<0.001*
PASAT-3^d^	n = 13	n = 10	n = 10	
Raw score (correct number/60)	34.15 ± 10.58 ^A^	47.60 ± 10.29^B^	52.00 ± 6.68^B^	<0.001*
Z-score	−2.67 ± 1.58 ^A^	−0.66 ± 1.54 ^A^	0 ± 1^B^	<0.001*
MSFC sum score	n = 10	n = 10	n = 10	
Z-score^e^	−6.72 ± 2.84 ^A^	−6.05 ± 4.8^A^	0 ± 0.57^B^	<0.001*

Abbreviations: EDSS, Expanded Disability Status Scale; FSS, Fatigue Severity Scale; IQR, interquartile range; MSFC, Multiple Sclerosis Functional Composite; MS, multiple sclerosis; n/a, not applicable or not available; NMOSD, neuromyelitis optica spectrum disorders; PASAT-3, Paced Auditory Serial Addition Test with a 3-second interstimulus interval; SD, standard deviation; T25-FW, Timed 25-Foot Walk Test; 9HPT-dominant, 9-Hole Peg Test of the dominant hand.

^A,B,C^The same character indicates no difference between groups, while a different character indicates a difference.

^a^A z-score is a standardized number, in this study representing how close a test result is to the mean of 10 healthy controls and expressed as a standard deviation. A negative z-score indicates that the test result was worse than the mean performance of the 10 healthy controls, and the value indicates the number of standard deviations.

^b^The T25-FW could not be performed in two patients with NMOSD because of limited physical ability, and was not completed in one patient with NMOSD because of physical fatigue.

^c^One subject with NMOSD was unable to perform the test on the non-dominant hand because of limited physical ability, so a value of 777 seconds was applied as suggested by the MSFC scoring manual.

^d^One patient with NMOSD and two with MS refused to perform the PASAT-3 test because they stated that they did not understand the principle of the test.

^e^The MSFC sum Z score was calculated for subjects who completed all tests by combining the three components into a single z score.

### MRI

#### Lesions

Lesion count and volume, and the quantitative T1 and MTR of the lesions were measured in each subject. The lesion count and volume were significantly higher in patients with MS than in those with NMOSD or healthy controls (Table 3). The lesions were mainly localized in the supratentorial regions of patients with MS, and in the infratentorial regions of patients with NMOSD. A few incidental, small and nonspecific lesions in the supratentorial deep WM were identified in four controls. The lesions in the patients with MS had a significantly higher measured T1 than those in patients with NMOSD or controls. Similarly, MS lesions had the lowest mean lesion MTR value, followed by NMOSD lesions. There was a correlation between mean T1 and mean MTR for the lesions in patients with NMOSD (Spearman’s rho = −0.800, *p* = 0.001) and for those of patients with MS (rho = −0.912, *p* < 0.001).

**Table 3:** Comparison of lesion load, T1 relaxation time and MTR among the NMOSD patients, MS patients and healthy controls.

	NMOSD (n = 14)	MS (n = 12)	Controls (n = 10)	P
**Lesion count** (median, IQR)	11^A^ (2.0–18.0)	74^B^ (33.5–112.5)	0^A^ (0.0–3.0)^#^	<0.001*
**Lesion volume (mm**^3^) (median, IQR)	242.4^A^ (60.7–696.0)	5217.0^B^ (2356.8–9488.2)	0^A^ (0–27.2)^#^	<0.001*
**T1 relaxation time (ms)** Mean ± SD	1869 ± 367^A^	2163 ± 268^B^	1511 ± 117^A^	0.003*
**MTR (%)** Mean ± SD	38.8 ± 6.5^A^	32.0 ± 4.6^B^	46.5 ± 3.8^C^	<0.001*

Abbreviations: IQR, interquartile range; MS, multiple sclerosis; MTR, magnetization transfer ratio; NMOSD, neuromyelitis optica spectrum disorders; SD, standard deviation.

^#^Among the 10 healthy controls, 4 had incidental findings of at least one nonspecific white matter lesion.

^A,B,C^The same character indicates no difference between groups, while a different character indicates difference.

In the patients with NMOSD, the mean lesion T1 was negatively correlated with cognition score (rho = −0.592, *p* = 0.033). In the patients with MS, lesion volume was positively correlated with disease duration, and negatively correlated with hand function score.

#### NAWM

**Figure 1:**
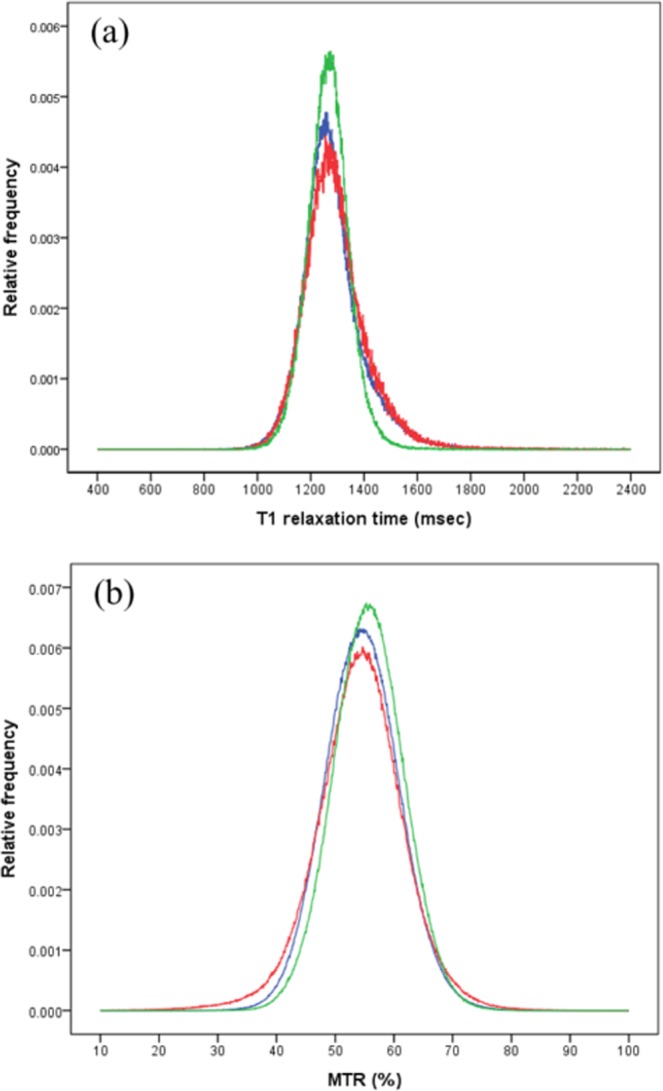
The averaged histograms of the normal-appearing white matter (NAWM) in neuromyelitis optica spectrum disorder (NMOSD; blue) patients, multiple sclerosis (MS; red) patients and healthy controls (green). **(a**) T1 relaxation time (T1): the average peak height in the T1 histogram of the NAWM of NMOSD patients was significantly lower than that in healthy subjects and did not differ from that in MS patients, reflected in the greater width of the NMOSD and MS histograms. The peak position was not statistically different among the three groups. **(b)** Magnetization transfer ratio (MTR): The average peak height in the MTR histogram of the NAWM of NMOSD patients was significantly lower than that in healthy subjects and did not differ from that in MS patients, reflected in the relative widths of these frequency histograms. The peak position was not statistically different among the three groups, but in MS patients, the 25^th^ percentile value for MTR was lower than in healthy participants.

The T1 and MTR were calculated for the NAWM and histogram metrics were used to asses this, to increase power by comparing the whole region of interest (ROI). The results are shown in Table 4, and averaged histograms are shown in Fig. 1. The heights of the peaks of both the T1 and MTR histograms of the NAWM of the NMOSD patients were significantly lower than those in the healthy subjects, but were not different from those of the patients with MS. The peak position was not statistically different among the three groups; however, in the patients with MS, the 25th percentile MTR value was lower than that for the healthy participants. This pattern indicates that there are focal areas of abnormality rather than diffuse abnormality within the voxels.

**Table 4:** Comparisons of the NAWM histogram metrics, as indexed by T1 relaxation time or MTR, among the NMOSD patients, MS patients and healthy controls.

	NMOSD (n = 14)	MS (n = 12)	Controls (n = 10)	P
**T1 relaxation time (mean ± SD)**				
Peak height (10^–3^ pixels)	5.9 ± 1.5^A^	5.6 ± 1.2^A^	6.8 ± 1.0^B^	0.067*
Peak position (ms)	1244 ± 43	1265 ± 47	1264 ± 25	0.915
25th percentile (ms)	1203 ± 40	1217 ± 31	1218 ± 27	0.990
50th percentile (ms)	1259 ± 49	1282 ± 37	1265 ± 23	0.604
75th percentile (ms)	1321 ± 64	1353 ± 55	1312 ± 22	0.204
**MTR (mean ± SD)**				
Peak height (10^-3^, pixels)	7.7 ± 1.5^A^	6.7 ± 1.3^A^	8.9 ± 1.0^B^	0.002*
Peak position (%)	55.8 ± 3.4	54.2 ± 3.2	56.2 ± 3.8	0.401
25th percentile (%)	51.5 ± 2.9 ^A^	49.3 ± 3.3^B^	52.5 ± 3.5^A^	0.081*
50th percentile (%)	55.4 ± 3.1	53.9 ± 2.9	55.7 ± 3.6	0.400
75th percentile (%)	59.1 ± 3.3	58.4 ± 3.0	58.9 ± 3.8	0.926

The mean values of five parameters for each group (the height and position of the histogram peak and the 25th, 50th and 75th percentile values) were included in the analysis.

Abbreviations: NMOSD, neuromyelitis optica spectrum disorders; MS, multiple sclerosis; MTR, magnetization transfer ratio; SD, standard deviation.

^A,B,C^The same character indicates no difference between groups, while a different character indicates a difference.

In the patients with MS, T1 histogram metrics were correlated with MSFC score, and MTR histogram metrics were correlated with leg function score using Spearman’s rank correlation coefficient (Table 5). No such correlations were noted in the patients with NMOSD.

**Table 5:** Correlation coefficients of MRI metrics of normal-appearing white matter, lesion parameters and clinical factors.

	Lesion			NAWM T1 relaxation time					NAWM MTR				
	Volume	Mean T1	Mean MTR	Peak height	Peak position	p25	p50	p75	Peak height	Peak position	p25	p50	p75
**NMOSD**													
Disease duration	−0.228	−0.104	0.009	0.320	−0.300	−0.283	−0.369	−0.413	0.205	0.384	0.501	0.426	0.340
EDSS	−0.206	−0.048	−0.170	−0.213	−0.327	−0.347	−0.191	−0.041	−0.183	0.052	0.141	0.177	0.219
FSS	−0.392	−0.332	0.513	0.612*	−0.173	−0.138	−0.330	−0.448	−0.144	0.443	0.415	0.429	0.416
MSFC-z score	0.454	0.021	0.112	0.022	0.514	0.590	0.436	0.239	0.106	−0.094	−0.175	−0.255	−0.312
T25-FW-z score	0.405	0.033	0.092	−0.019	0.479	0.524	0.390	0.220	−0.014	0.030	−0.050	−0.090	−0.113
9HPT-z score	−0.299	0.415	−0.134	0.065	−0.199	−0.189	−0.168	−0.137	0.120	0.433	0.385	0.374	0.308
PASAT3-z score	0.325	−0.592*	0.332	0.093	0.048	−0.077	−0.139	−0.198	−0.003	−0.031	0.040	0.031	0.059
Lesion volume	1	−0.080	−0.173	−0.263	0.715**	0.604*	0.611*	0.516	0.147	−0.584*	−0.542*	−0.587*	−0.553*
Lesion mean T1	−0.080	1	−0.800**	0.169	−0.006	0.057	0.017	−0.034	0.473	−0.014	0.044	−0.083	−0.216
Lesion mean MTR	−0.173	−0.800**	1.000	0.184	−0.254	−0.237	−0.293	−0.274	−0.404	0.307	0.216	0.312	0.391
**MS**													
Disease duration	0.639*	0.054	−0.266	0.498	−0.029	−0.044	−0.103	−0.161	0.347	−0.597*	−0.301	−0.499	−0.591*
EDSS	0.375	0.377	−0.211	−0.204	0.202	0.169	0.374	0.453	0.007	−0.195	−0.137	−0.123	−0.082
FSS	0.190	−0.126	0.188	−0.019	−0.113	0.029	0.219	0.356	−0.012	−0.034	−0.062	0.028	0.132
MSFC-z score	−0.465	−0.134	−0.014	0.634*	−0.886**	−0.710*	−0.813**	−0.842**	−0.193	0.512	0.446	0.482	0.505
T25-FW-z score	−0.498	−0.236	0.120	0.310	−0.253	−0.329	−0.568	−0.638*	0.057	0.644*	0.588*	0.606*	0.525
9HPT-z score	−0.828**	−0.414	0.520	−0.088	−0.204	−0.317	−0.452	−0.468	−0.075	0.299	0.231	0.243	0.219
PASAT3-z score	−0.174	0.014	−0.009	0.722*	−0.406	−0.248	−0.303	−0.318	0.529	0.320	0.377	0.251	0.152
Lesion volume	1	0.217	−0.467	0.333	0.373	0.267	0.288	0.210	0.463	−0.299	−0.057	−0.229	−0.367
Lesion mean T1	0.217	1	−0.912**	−0.351	0.003	0.105	0.307	0.431	−0.331	−0.071	−0.205	−0.085	0.051
Lesion mean MTR	−0.467	−0.912**	1.000	0.141	−0.037	−0.053	−0.204	−0.300	0.148	0.060	0.119	0.061	−0.007
**Healthy controls**													
FSS	−0.572			0.047	−0.060	0.049	0.024	0.022	−0.082	−0.046	−0.038	−0.028	−0.020
MSFC-z score	0.244			0.336	−0.106	−0.082	−0.236	−0.398	−0.054	0.010	−0.044	−0.045	−0.051
T25-FW-z score	0.441			0.444	0.035	0.158	0.016	−0.155	0.322	−0.506	−0.509	−0.527	−0.543
9HPT-z score	−0.093			0.452	0.270	0.254	0.106	−0.184	0.168	0.246	0.279	0.243	0.197
PASAT3-z score	0.066			−0.324	−0.486	−0.552	−0.524	−0.338	−0.582	0.276	0.155	0.208	0.259
Lesion volume	1			0.060	−0.128	−0.202	−0.270	−0.337	0.081	−0.207	−0.285	−0.283	−0.287

*Correlation significant at the 0.05 level (2-tailed). **Correlation significant at the 0.01 level (2-tailed).

Abbreviations: EDSS, Expanded Disability Status Scale; FSS, Fatigue Severity Scale; MSFC, Multiple Sclerosis Functional Composite; MS, multiple sclerosis; MTR, magnetization transfer ratio; NMOSD, neuromyelitis optica spectrum disorders; PASAT-3, Paced Auditory Serial Addition Test with a 3-second interstimulus interval; p25, 25^th^ percentile; p50, 50^th^ percentile; p75, 75^th^ percentile; T1, T1 relaxation time; T25-FW, Timed 25-Foot Walk Test; 9HPT, 9-Hole Peg Test.

#### Lesion volume and NAWM

In the patients with NMOSD, both the T1 and MTR histogram metrics of the NAWM were correlated with lesion volume. No such correlation was found in the patients with MS.

## Discussion

This study used high-resolution 7T MRI to compare the T1 and MTR of intracranial lesions and NAWM among NMOSD patients, MS patients, and healthy controls. Compared to MS lesions, the NMOSD lesions showed less obvious tissue changes. Regarding the NAWM, NMOSD patients had a lower histogram peak height for both T1 and MTR compared to controls. None of the histogram metrics were significantly different from those of the patients with MS although there was a trend towards the NMOSD measures differing less from those of the controls compared to the MS patients, similar to the trends in changes observed in the lesions. The decreased peak height of NAWM in both the MS and NMOSD patients indicated that at least some of the NAWM in the patients with NMOSD was abnormal, as has been shown in patients with MS. The histogram metrics for the NAWM were correlated with lesion volume in the patients with NMOSD, but not in those with MS. Taken together, our study suggests that the NMOSD lesions had less myelin damage than the MS lesions, and that the NAWM in patients with NMOSD is abnormal, where the abnormal changes were correlated with lesion volume.

T1 is an intrinsic biophysical property of tissue, and is primarily determined by water binding to macromolecules, including myelin. In MS, increased T1 of the WM is highly associated with reduced structural integrity, and black holes (the darkest area on T1 weighted images) have the highest T1^13^. An elevated T1 correlates with a loss of myelin, axons^14^ and increased free water, which to some extent reflects gliosis by virtue of the increase in water protons^17^. Magnetization transfer (MT) imaging measures proton exchange between free protons and macromolecular bound protons and provides a relatively accurate measurement of the myelin content^12,18^. Our MS results for the lesions were similar to those observed in previous studies, reflecting decreased myelin content and tissue integrity, which can be due to demyelination or incomplete remyelination^12,15^.

Our results showed an abnormal mean MTR, but not T1, in the lesions of patients with NMOSD. However the deviation from normal was smaller than in the patients with MS. Our findings reject the first hypothesis. In contrast to previous histopathological findings of focal necrosis in autopsied patients with fulminant NMO^6,7^, the patients with NMOSD in the current study had fewer destructive lesions in the brain overall than those with MS. One possible explanation for this is that our patients with NMOSD benefited from effective treatment such as plasma exchange, immunosuppressants or B cell depletion therapy. Another possibility is lesion heterogeneity, as the standard deviation of lesion T1 and MTR in NMOSD patients, was as wide as that in MS patients, and several types of lesions have been reported in NMOSD^6,19^. The histopathological hallmark of the active lesions of NMOSD patients with AQP4 antibodies is loss, or decreased staining, of AQP4 protein, and an absence of astrocytes or loss of astrocyte distal processes^19^, thus disrupting the cerebrospinal fluid (CSF)-brain barrier and blood-brain barrier, and leading to necrosis^6,20^. Nevertheless, the early active lesions in NMOSD patients showed relative preservation of myelin compared to those of MS patients^19^. However, these changes may not contribute sufficient water to account for the observed changes measured by T1, because other alterations, such as infiltration of eosinophils or granulocytes, have also been observed^6^. Furthermore, the patients with MS in this study had a relatively longer disease course than those with NMOSD; as such, tissue destruction of chronic lesions may be more obvious in MS. Overall, our results seem to be compatible with the known pathological characteristics of primary astrocytopathy occurring in NMOSD, and primary demyelination occurring in MS^21^.

In this study, histogram metrics were used to assess the NAWM to provide maximum power to detect changes. The histogram metrics were statistically indistinguishable between the NMOSD and MS patients, rejecting the second hypothesis. Regarding the T1 histogram of patients with PPMS, the peak height decreased and the peak position increased; therefore, the histogram metrics shifted rightwards over 2 years^22^. If myelin was reduced in the NAWM, the histogram metrics would shift leftwards, such that the peak height and peak position would both decrease. Previous studies have shown that abnormalities in MS NAWM are subtype-dependent^23^: SPMS shows changes in both the peak position and height of NAWM histograms, whereas RRMS and PPMS show decreased height only^23–25^. Since our study included more patients with RRMS and PPMS, a change in NAWM peak location was not observed. However, our patients with MS showed a decrease in the peak height of both the T1 and MTR NAWM histograms, suggesting a decreased proportion of normal WM. The results also revealed a decreased proportion of normal WM in patients with NMOSD compared to controls.

The results of the current study are in line with previous DTI studies of changes in the microstructural diffusivity of WM tracts in optic tracts, medulla oblongata, and corticospinal tracts^8,10,11^, and provide further evidence of reduced myelin content in the NAWM of NMOSD patients, based on myelin content-specific MT imaging. DTI, a tool for investigating the microstructural diffusivity of WM tracts, revealed abnormalities in optic tracts, the corpus callosum, the medulla oblongata, and corticospinal tracts in NMOSD patients^8,10,26–28^. DTI at 3T showed greater diffusivity changes in the NAWM of 93 patients with NMOSD compared to 43 healthy controls, and the extent of DTI-detectable NAWM changes was less than that in 53 patients with MS^11^. Measuring changes in the diffusion of water in directional structures, such as nerve bundles, in the brain provides insights into the structural integrity, in terms of myelin and axonal content, of MS patients^29,30^. However, NAWM axons in patients with NMOSD seemed not to be affected, as measured by MR spectroscopy at 1.5T or 3T field strength^16,31,32^. Using MTR quantitative imaging at 1.5T field strength to evaluate the NAWM of NMO patients, two earlier studies did not find altered MTR parameters^8,33^. However, using 7T MRI, we found evidence of changes in myelin in the NAWM of patients with NMOSD.

The abnormal findings in the NAWM of the patients with NMOSD noted in this study appear to be robust, because they were shown by two different MRI techniques based on different physical principles. MTR at 1.5T failed to distinguish the NAWM between NMOSD and controls, in terms of either mean values or peak heights^8,33^. However, using 7T MRI, we found evidence of abnormal myelin and water content in NAWM, and this change was correlated with lesion volume mainly localized in the infratentorium. We assume that we were able to detect MTR changes because of the ultra-high field strength. It is possible that the difference in spatial resolution between our study and those using lower field strengths made it easier to observe smaller changes in NAWM herein. This could be verified by repeating the 7T scanning at the spatial resolution used in previous lower field strength studies. Histopathological studies to clarify the current findings pertaining to the NAWM are also warranted. Nevertheless, the current study did not find an association between cognition and MRI parameters. This may be attributed to the small number of patients and the mixture of NMOSD subtypes.

Several limitations of the current study deserve discussion. First, the number of patients included was small. We intentionally included different MS subtypes, based on prior knowledge of the likelihood of NAWM abnormalities, to match for disability levels. However, the mixture of phenotypes may have introduced bias, including in the cognition measures. Second, lesion detection was performed using MT_sat_ (MTR based on proton- and T2*-weighted imaging with presaturation to water) images, which might not be as accurate as 3T fluid-attenuated inversion recovery (FLAIR) images. However, we previously showed that 7T-MT_sat_ sequences detected most of the WM lesions identified by 3T-FLAIR sequences, and missed only approximately 1.5% of small lesions^34^; hence, patients do not need to undergo both 7T and 3T scans on the same day. Third, the temporal lobes were not all included because the signal loss caused by inhomogeneity in the applied transmit radiofrequency (RF) field at high field strength was more pronounced in these regions.

Furthermore, the NAWM map included supratentorial, basal ganglia and infratentorial regions, where pathology of the basal ganglia and infratentorial brain might be different from that of supratentorial areas; further studies are needed to clarify this. Nevertheless, the current study was strengthened by the piloting of two quantitative methods based on 7T MRI, used to compare two groups of patients matched for demographic characteristics and level of disability.

## Conclusions

Our analysis of T1 and MTR values suggested that the lesions in the NMOSD patients had decreased myelin signal, but the changes therein were less pronounced compared to those in the patients with MS, in turn suggesting that they were less myelin-destructive. 7T MRI demonstrated a decreased peak height of NAWM histograms in NMOSD patients compared to controls. However, the histogram metrics were statistically indistinguishable from those in the patients with MS, suggesting that at least part of the NAWM in the patients with NMOSD was indeed abnormal, with reduced myelin, which had not been previously observed using MRI at a lower field strength.

## Methods

This study was approved by our local Research Ethics Committee (REC), and by the relevant National Health Service (NHS) Research & Development (R&D) department (REC reference: 13/EM/0080). The research was performed in accordance with relevant guidelines and regulations. Informed consent was obtained from all participants.

### Patients

Consecutive patients with NMOSD referred to the Neuroimmunology Clinic of University Hospital Nottingham, between July 2013 and November 2014, were enrolled at the Sir Peter Mansfield Imaging Centre (SPMIC) at the University of Nottingham. Patients were eligible if (1) they had a diagnosis of NMOSD^4^ or MS^35^ as assessed by specialists, and (2) they were clinically stable, fully conscious and orientated. Age- and sex-matched patients with MS were recruited simultaneously. We used posters to recruit healthy volunteers, who were interviewed by a neurologist. The exclusion criteria included claustrophobia, metal objects or devices implanted in the body, and pregnancy. The patients were assessed using the EDSS, MSFC, and FSS. Healthy volunteers were only assessed with the MSFC and FSS.

### MRI image acquisition

All participants underwent MRI scanning using a 7T Philips Achieva (Philips Medical Systems, Best, The Netherlands) with whole-body gradients. The scanner was equipped with a 32-channel head-only parallel imaging sensitivity encoding (SENSE) receiver coil and a head-only volume quadrature RF coil (Nova Medical, Inc., Wilmington, MA, USA). The parameters of the MRI sequences are detailed in Supplementary Table 2, and the techniques used have been detailed previously^36–39^. Briefly, whole brain three-dimensional (3D) phase-sensitive inversion recovery (PSIR) images (spatial resolution = 0.6 × 0.6 × 0.6 mm^3^) were acquired and segmented to obtain a WM mask^39^. Then, 3D magnetization-prepared rapid gradient echo (MPRAGE) sequences with seven inversion times were acquired (spatial resolution = 1.25 × 1.25 × 1.25 mm^3^) and processed to produce quantitative measurements of T1^38^. In addition, MT images were acquired for quantitative measurement of MTRs derived from two raw images: MT_nosat_ (proton- and T2*-weighted imaging with no presaturation), and MT_sat_ (presaturation to water) (spatial resolution = 0.6 × 0.6 × 0.6 mm^3^)^36,37^. MT_sat_ imaging was also used to identify lesions. The scanning protocol included a B1 map to correct the MT images for the effects of B1 inhomogeneity.

### Post-processing

Firstly, we obtained a lesion mask for each subject. Lesions were visually identified and outlined on MT_sat_ using a semi-automated seed-growing technique, implemented in the in-house NeuRoi software (http://www.nottingham.ac.uk/scs/divisions/clinicalneurology/software/neuroi.aspx). The lesions were identified in all image slices, which covered the supratentorium, midbrain, rostral pons, and part of the cerebellum. The total volume of these lesions was then calculated, and a binary lesion mask was generated using NeuRoi.

Secondly, we obtained the NAWM mask. Initially, a WM probability map was segmented from PSIR images using an automatic segmentation process, implemented in Statistical Parametric Mapping software (SPM8; http://www.fil.ion.ucl.ac.uk/spm/), with a threshold value of 0.95 to generate a binary WM mask. Next, a lesion-free WM mask was generated by applying the lesion masks. Subsequently, the lesion-free WM mask was eroded by three voxels to exclude any partial volume effects at the grey matter-WM junctions, lesion-WM or CSF-WM borders to generate the NAWM mask^38^.

Thirdly, T1 and B1-corrected MTR maps were generated based on the raw images for each subject on a pixel-by-pixel basis. The T1 map was generated using formula (1)^40,41^:

1 $${M}_{0}=M(1-\alpha \,exp(\frac{-T1}{T1}))$$

The MTR map was generated using formula (2):

2 $${\rm{MTR}}=\frac{(M{T}_{nosat}-M{T}_{sat})}{M{T}_{nosat}}$$

**Figure 2:**
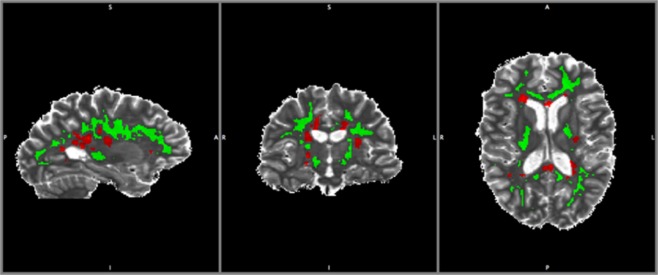
Illustration of the normal-appearing white matter (NAWM) mask. A lesion mask (red) and a NWAM mask (green) were fitted to the T1 map of a patient with MS.

Finally, the T1 and B1-corrected MTR maps were combined with the lesion and NAWM masks, to yield lesion and NAWM maps for each subject (Fig. 2). All voxels of NAWM with an MTR of less than 10% were excluded from the analysis to effects of image noise and CSF signals^42^. All of the acquired images for a given subject were registered to the same space using FLIRT in FSL (available at www.fmrib.ox.ac.uk/fsl).

We compared lesion properties based on the mean values of the lesion maps. To detect subtle changes in NAWM, histogram metrics of the NAWM maps derived from each subject were normalized to the total voxel count to eliminate any effect of differences in NAWM volume among subjects. The bin size for NAWM is 1 ms for T1 and 0.1% for MTR. Five histogram parameters were extracted: the height and position of the histogram peak and the 25th, 50th and 75th percentile values.

### Power calculation

We assumed that the peak height and position of the NAWM histogram of NMOSD patients were significantly different from those of healthy controls, because DTI imaging at 3T revealed diffusivity changes along optic and cortico-spinal tracts^10,11^. Because no quantitative T1 or MTR data of NAWM at 7T in NMOSD patients are available, we performed a pre-protocol power calculation based on data derived from a previous MS study^38^. We calculated the T1 of WM and peak heights for 9 patients with RRMS and 11 healthy controls. We predicted that the difference in NAWM histograms between patients with NMOSD and controls would be 40% of that between MS patients and controls. To determine the actual difference using a 2-tailed t-test, 10 subjects per group were required for 85% power at a significance level of 0.05, with a medium effect size.

An anticipated difficulty for 7T MRI studies of NMOSD patients is recruitment of a sufficient number of patients to obtain acceptable quality images for analysis. Some patients were too severely disabled (e.g. bed-ridden, limb spasticity) or lived a long distance from the 7T suite at the University of Nottingham. We estimated the number of MS and NMOSD patients in England using the Clinical Practice Research Datalink (CPRD), which is a database focused on primary care in the UK, and linked to secondary care in England. The estimates showed that NMOSD is very rare in England, with a prevalence rate in 2012 of 3.7 per 100,000 people (Chou *et al*., unpublished). The prevalence of MS is 70 times higher than that of NMOSD. Hence, the recruitment target for each group was 10 subjects.

### Statistical analysis

Comparisons of subgroups were performed using Fisher’s exact test for categorical data, the Mann-Whitney U test for ordinal (clinical rating scale) data, and ANOVA for continuous data. Multiple comparisons were conducted using the least significant difference (LSD) test, to identify which groups were different when ANOVA showed statistical differences among groups. Statistical significance was established with a cut-off level of 0.1. To assess correlations between clinical parameters and MRI-derived parameters (Table 5), Spearman’s rank correlation coefficient (rho) was used. All statistical tests were two-tailed, and all calculations were performed using SAS software (version 9.4; SAS Institute, Cary, NC, USA); correlation coefficients were calculated using the SPSS software package (version 22; IBM Corp, Armonk, NY, USA).

## Supplementary information

Supplementary Table 1 Imaging Sequences. Supplementary Table 2. Clinical information informing the diagnosis of NMOSD in each patient.

## Data Availability

All data generated or analysed during this study are included in this article or the Supplementary Information Files.
