# Supplementary material for: Reduced Myelin Signal in Normal-appearing White Matter in Neuromyelitis Optica Measured by 7T Magnetic Resonance Imaging
Source: Sci Rep. 2019 Oct 7;9:14378. doi: 10.1038/s41598-019-50928-0 (PMC6779889; doi:10.1038/s41598-019-50928-0)
Supplement: Supplementary file 1 — Supplementary Table 1 Imaging Sequences. Supplementary Table 2. Clinical information informing the diagnosis of NMOSD in each patient. [file 41598_2019_50928_MOESM1_ESM.docx]

**Reduced Myelin Signal in Normal-appearing White Matter in Neuromyelitis Optica Measured by 7T Magnetic Resonance Imaging**

I-Jun Chou, MD, PhD^1-3^, Radu Tanasescu, MD, PhD^1,4^, Olivier E. Mougin, PhD^5^, Penny A. Gowland, PhD^5^, Christopher R. Tench, PhD^1^, William P. Whitehouse, MD^2^, Bruno Gran, MD, PhD^1^, Esmaeil Nikfekr, MD, PhD^1^, Basil Sharrack, MD, PhD^6^, Gordon Mazibrada, MD^7^, Cris S. Constantinescu, MD, PhD^1^^*^

**Author Affiliations:**

^1^Division of Clinical Neuroscience; ^2^Division of Academic Child Health, School of Medicine, University of Nottingham, Nottingham, UK

^3^Division of Paediatric Neurology; Chang Gung Children’s Hospital at Linkou, Chang Gung University College of Medicine, Taoyuan, Taiwan

^4^Division of Neurosciences, University of Medicine and Pharmacy Carol Davila, Department of Neurology, Colentina Hospital, Bucharest, Romania

^5^Sir Peter Mansfield Imaging Centre, School of Physics and Astronomy, University of Nottingham, England, UK

^6^Department of Neurology, University of Sheffield, Sheffield, UK

^7^Department of Neurology, Queen Elizabeth Hospital Birmingham, Birmingham, UK

**Supplementary information**

| **Parameters** | **MT imaging** | **T1-MPRAGE imaging** | **PSIR imaging** |
| --- | --- | --- | --- |
| Data acquisition | 3D, magnetization transfer prepared TFE | 3D, magnetization prepared (inverted) TFE | 3D, magnetization prepared (inverted) TFE with two readouts |
| Repetition time/echo time (ms) | 12/6.4 | 13/6 | NA |
| Inversion time (ms) | NA | 7 sets (153, 303, 503, 803, 1203, 1803, 2503) | 2 sets (780 and 2380) |
| Shot-to-shot interval (s) | 10 | 8 | 5 |
| TFE factor | 450 | 240 per inversion | NA |
| Flip angle (degrees) | 8 | 8 | 8 |
| Field of view (mm^3^) | 192x180x120 | 200x180x120 | 200x181x120 |
| Resolution (mm) | 0.6x0.6x0.6 | 1.25x1.25x1.25 | 0.6x0.6x0.6 |
| Matrix dimension | 320x320x200 | 160x160x96 | 336x336x200 |
| Additional information | Two images: (1) MT_nosat_ was acquired with no presaturation, (2) MT_sat_ was acquired with presaturation 20 off-resonance pulses (sinc pulses) with a bandwidth of 300 Hz and off-resonance by 1.0 kHz (-3.4 ppm), 20 ms between each pulse. | Seven images from the inversion recovery | Two images from the inversion recovery reconstructed together |
| Acquisition time (min: s) | 12:55 | 13:53 | 11:35 |
| **Supplementary Table 1 Imaging Sequences.** Abbreviations: MT, Magnetization transfer; MPRAGE, magnetization-prepared rapid gradient echo; PSIR, phase-sensitive inversion recovery; 3D, three-dimensional; TFE, turbo field echo. | | | |

| **Patient** | **Clinical symptoms** | **Autoantibodies to AQP4 or MOG** | **Comorbidity or other autoantibody** | **Relapse** |
| --- | --- | --- | --- | --- |
| 1 | Recurrent LETM | AQP4 Ab+ |  | + |
| 2 | ON, LETM | AQP4 Ab+ |  | + |
| 3 | ON, LETM | AQP4 Ab+ |  | + |
| 4 | ON, LETM | AQP4 Ab+ | pANCA+ | + |
| 5 | ON, LETM | Seronegative for APQ4 Ab |  | + |
| 6 | ON, LETM | NMO-IgG+ | ANA+ | + |
| 7 | ON, LETM | seronegative |  | + |
| 8 | Recurrent LETM | Seronegative for AQP4 Ab |  | + |
| 9 | Recurrent LETM | NMO-IgG+ |  | + |
| 10 | ON, LETM | Seronegative for AQP4 Ab |  | + |
| 11 | ON, LETM | AQP4 Ab+ | SLE, Sjögren syndrome | + |
| 12 | ON, LETM | seronegative |  | + |
| 13 | Recurrent LETM | AQP4 Ab+ |  | + |
| 14 | ON, LETM | MOG Ab+ |  | + |

**Supplementary Table 2. Clinical information informing the diagnosis of NMOSD in each patient.** Abbreviations: ANA, antinuclear antibodies; AQP4 Ab, autoantibodies against aquaporin-4; MOG Ab, antibodies against myelin oligodendrocyte glycoprotein; NMO, neuromyelitis optic; NMOSD, neuromyelitis optica spectrum disorders; ON, optic neuritis; pNACA, perinuclear anti-neutrophil cytoplasmic antibodies; SLE, Systemic lupus erythematosus; LETM, longitudinally extensive transverse myelitis.
